# Differentiated cultures of an immortalized human neural progenitor cell line do not replicate prions despite PrP^C^ overexpression

**DOI:** 10.1080/19336896.2023.2206315

**Published:** 2023-05-02

**Authors:** Jessy A. Slota, Xinzhu Wang, Diana Lusansky, Sarah J. Medina, Stephanie A. Booth

**Affiliations:** a One Health Division, National Microbiology Laboratory, Public Health Agency of Canada, Winnipeg, Manitoba, Canada; b Department of Medical Microbiology and Infectious Diseases, Faculty of Health Sciences, University of Manitoba, Winnipeg, Manitoba, Canada; c Tanz Centre for Neurodegenerative Diseases, University of Toronto, Toronto, Ontario, Canada

**Keywords:** Creutzfeldt-Jakob disease, human neural progenitor cells, *in vitro* models, neurogenesis, prion disease, prion models, Scrapie

## Abstract

Prions are misfolded proteins that accumulate within the brain in association with a rare group of fatal and infectious neurological disorders in humans and animals. A current challenge to research is a lack of *in* *vitro* model systems that are compatible with a wide range of prion strains, reproduce prion toxicity, and are amenable to genetic manipulations. In an attempt to address this need, here we produced stable cell lines that overexpress different versions of PrP^C^ through lentiviral transduction of immortalized human neural progenitor cells (ReN VM). Differentiated cultures made from the neural progenitor cell lines overexpressed PrP^C^ within 3D spheroid-like structures of TUBB3^+^ neurons and we observed evidence that PrP^C^ modulates formation of these structures, consistent with PrP^C^’s role in neurogenesis. However, through repeated measurements of amyloid seeding activity in 6-week time course experiments, we failed to observe any evidence of prion replication within the differentiated ReN cultures following challenge with four prion isolates (human sCJD subtypes MM1 and VV2, and rodent adapted scrapie strains RML and 263K). We attributed amyloid seeding activity detected within the cultures to residual inoculum and concluded that PrP^C^ overexpression was insufficient to confer permissiveness of ReN cultures to prion infection. While our ReN cell prion infection model was unsuccessful, additional efforts to develop cellular models of human prion disease are highly warranted.

## Introduction

Prion diseases are rare infectious neurological disorders that afflict humans and animals, and are characterized by long pre-clinical incubation periods prior to rapidly progressive neurocognitive decline culminating in death [1,2]. The disease causing agent is thought to comprise of misfolded prion proteins (PrP^Sc^) that are deposited within the brain as plaques in association with neuropathological changes [3]. Accumulation and spread of prions are mediated by prion replication whereby PrP^Sc^ recruits and converts the host-encoded cellular prion protein (PrP^C^) into the disease-associated conformation [4]. Prion diseases are diverse with widely varying clinical signs and symptoms encoded by prion strains – defined as infectious isolates with distinct pathological features and are thought to adopt unique 3D conformations of misfolded PrP^Sc^ [5,6].

In the laboratory, prion infection is usually achieved through intracerebral inoculation of mice *in vivo* followed by monitoring of clinical signs over several months to years, faithfully recapitulating the disease seen in humans. However, *in vivo* models are long, laborious, costly, and wildtype mice are often infected with synthetic prion isolates that have been adapted to the host through repeated serial passaging [7]. Synthetic mouse-adapted prion strains are needed because of transmission barriers that are mainly attributed to differences between the amino acid sequence of host-encoded PrP^C^ and PrP^Sc^ present in the inoculum [4,6]. As such, specialized transgenic mice that express the PrP^C^ version of the natural host are often required to overcome transmission barriers and study naturally occurring prion isolates [8]. Alternatively, bank voles are considered a ‘universal prion acceptor’ because they are susceptible to a wide range of prion isolates including those from humans [9]. Nonetheless, *in vivo* mouse and other animal models enable correlation of neuropathological changes with onset of clinical signs and are considered the ‘gold standard’ prion bioassay. In contrast, development of *in vitro* models for prion infection has proven much more challenging.

A lack of accessible and useful *in vitro* models of prion infection has hampered progress towards identifying links between prion replication and toxicity, screening putative therapeutics, and characterizing prion strain diversity. This is particularly true for human prion isolates that have been notoriously difficult to culture. Indeed, despite numerous efforts over the years, an immortalized neuron-like cellular model has never successfully replicated naturally occurring human prion isolates [10]. Certain cell culture systems that propagate prions are often used to examine PrP^Sc^ trafficking, but are mostly limited to isolates of mouse-adapted scrapie and usually do not reproduce prion toxicity [11,12]. In contrast to immortalized cell cultures, primary neuronal cultures can be used in the study of acute prion toxicity but do not have the longevity required for prion replication [13]. Cultured organotypic brain sections have proven to be one of the best *in vitro* models of prion infection because following several weeks of PrP^Sc^ replication, they reproduce the pathological hallmarks of the disease [14–16]. However, this system requires a steady supply of live animals, specialized equipment and a high level of technical expertise, reducing accessibility to researchers. Thus, there is much room for improvement with respect to *in vitro* models of prion infection.

Cultures derived from differentiated neural progenitor cells have successfully been used to replicate prions *in vitro* and in some cases have reproduced prion toxicity [17–24]. We therefore hypothesized that overexpressing different PrP^C^ versions in neural progenitor cells prior to differentiation would serve as a useful model for prion infection. An interchangeable system like this could permit characterization of a wide range of prion strains and might serve as a platform to examine the effects of targeted genetic manipulations on prion infection. Accordingly, here we describe, the lentivirus-delivered expression of PrP^C^ transgenes in an immortalized human neural progenitor cell line (ReN VM) that had been ablated for its endogenous PrP^C^ expression (ReN *PRNP*^−/−^ cells). We chose ReN VM cells because they have previously been shown to replicate misfolded amyloid-β and phosphorylated tau, serving as a model of Alzheimer’s disease [25–28]. ReN cell lines were produced that express human, hamster and mouse version of PrP^C^ and were differentiated into TUBB3^+^ neurons and GFAP^+^ astrocytes prior to challenge with four prion isolates. Prion replication was assessed through repeated measurements of amyloid seeding activity in 6-week time course experiments. Strikingly, these ReN cell-derived cultures were incapable of replicating multiple prion isolates despite the high level of PrP^C^ expression.

## Results

### Characteristics of ReN neural progenitor-derived cultures

**Figure 1.**
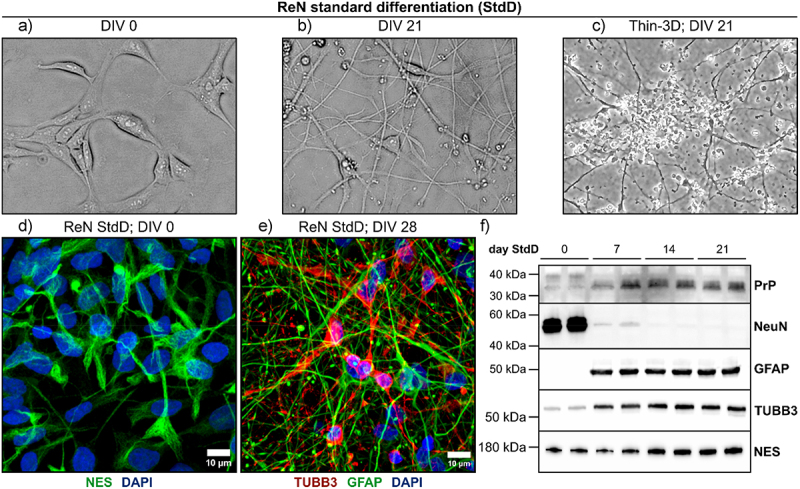
In vitro characteristics of ReN human neural progenitor-derived cultures. (a) and (b) ReN neural progenitor cells differentiate into cultures with neuronal morphology over 21 DIV. (c) ReN cells can also be differentiated as thin 3D cultures embedded within Matrigel matrix. (d) Representative Z-stacked projection of ReN neural progenitor cells stained with Nestin (NES; green) before differentiation. (e) Representative Z-stacked projection of neurons and astrocytes in thin-3D ReN cells at day 28 post-standard differentiation. Neurons were stained with beta-III-tubulin (TUBB3; red), astrocytes were stained with glial fibrillary acidic protein (GFAP; green) and nuclei were counterstained using DAPI (Blue). Immunofluorescence images were acquired using the 63X oil immersion objective of a Zeiss LSM 700 instrument (scale bar = 10 µm). (f) ReN cells express markers of neurons (TUBB3) and astrocytes (GFAP), neural progenitors (NES) and PrP^C^ throughout standard differentiation. NeuN expression was also detected in ReN lysate at day 0 StdD.

We explored the use of the ReN VM human neural progenitor cell line as a platform for modelling replication of human prions *in vitro*. First, we characterized the wildtype ReN VM cell line (denoted here as ReN WT) that possesses the intact human *PRNP* gene with the MV polymorphism at codon 129. Upon growth factor removal, ReN progenitor cells lose their ability to proliferate and instead differentiate into glial and neuronal cell types, coinciding with morphological changes as seen in the cultures [29–32]. When differentiated as 2D monolayers, ReN cells take on a predominantly neuronal morphology with rounded cell bodies and increasing numbers of neuronal projections over at least 3 weeks *in vitro* (Figure 1a,b). By embedding ReN cells within Matrigel, they can also be differentiated as thin-3D cultures. We found ReN cells within these thin 3D cultures to self-arrange into spheroid-like clusters of cells (Figure 1c), similar to 3D neurospheroids produced by culturing ReN cells in low-attachment round-bottomed plates [33] or microwell chips [34]. The morphology of ReN progenitor cells was confirmed by immunofluorescence staining of NES at day 0 of differentiation (Figure 1d), while the morphology of ReN neurons and astrocytes was examined by staining for TUBB3 and GFAP at day 28 post-differentiation (Figure 1e).

To examine the kinetics of ReN cell differentiation over time, we also tracked protein expression through western blotting of neuronal and astrocytic markers in lysate collected at days 0, 7, 14 and 21 post-differentiation (Figure 1f). As anticipated, the expression of neuronal marker TUBB3 and astrocytic marker GFAP increased by day 7 *in vitro*. We also found the expression of PrP^C^ to increase steadily upon differentiation, starting at day 7 *in vitro*. Unexpectedly, we observed weak expression of NeuN (mature neuron marker) by the ReN progenitor cells at day 0, and this expression was quickly lost upon differentiation. We also failed to detect expression of synaptic marker SYN1, or oligodendrocyte marker OLIG2 by ReN cells via western blotting at any time-point post differentiation (supplementary figure S1). Taken together, while differentiated ReN cells expressed markers of neurons and astrocytes, in our hands they did not fully mature into neurons that express markers of synaptic signalling.

### Creation of ReN cell lines that overexpress PrP^C^ in TUBB3^+^ neurons following differentiation

**Figure 2.**
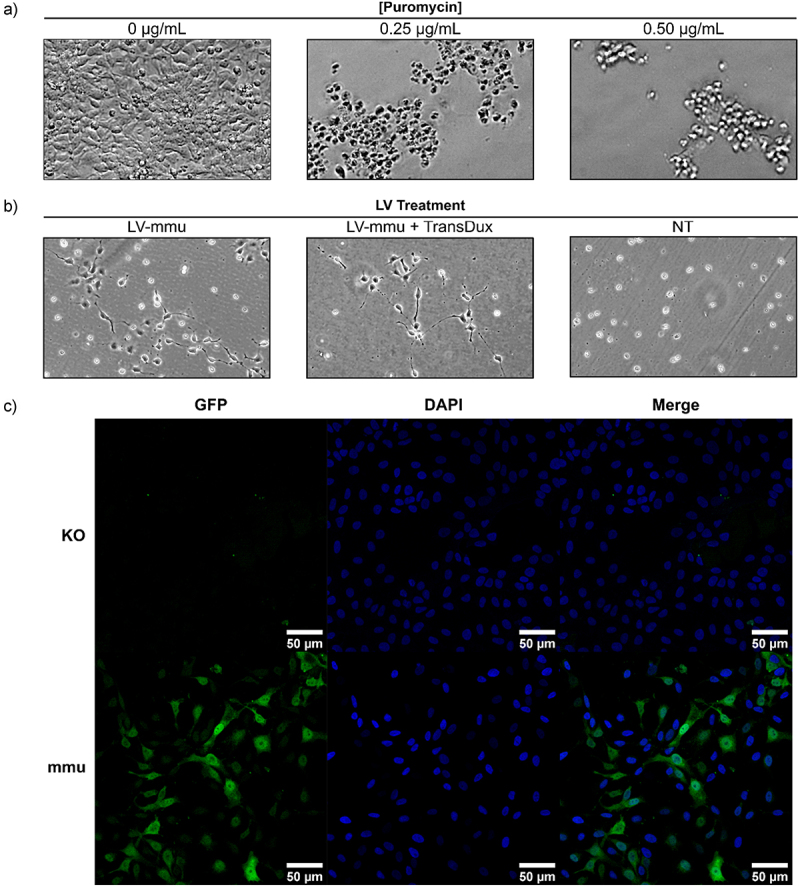
Enrichment of GFP expressing transduced ReN cells using puromycin antibiotic selection. (a) Kill curve with puromycin antibiotic on non-treated ReN cells revealed a concentration of 0.5 µg/mL was sufficient to kill all cells after 48 hours. (b) Lentiviral-transduced ReN cells can be selected with puromycin antibiotic treatment. ReN cells were treated with 1 μL of mouse PrP^C^ expressing lentiviral prep (LV-prep) for 72 hours, with or without TransDux enhancer, at which point the cells were treated with 0.5 μg/mL puromycin. Images were taken with a phase contrast microscope 3 days following puromycin treatment. (c) GFP expression in ReN cells before and after lentiviral transduction. Cells were fixed and stained with DAPI before imaging for GFP via fluorescence microscopy. Images were acquired using the 40X oil objective of a Zeiss LSM 700 instrument (scale bar = 50 µm).

**Figure 3.**
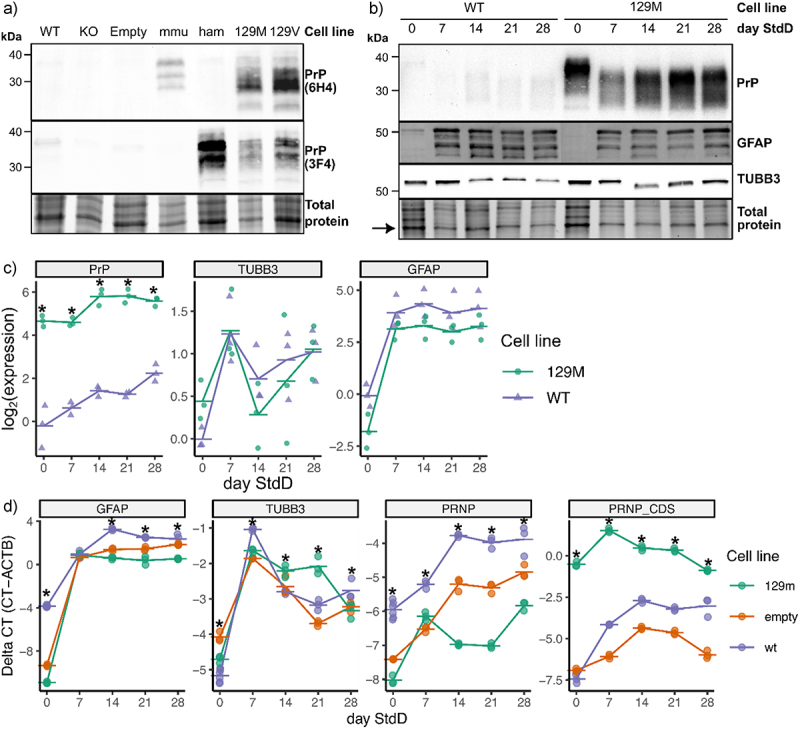
Lentiviral-transduced ReN cell lines overexpress PrP^C^ and differentiate into neurons and astrocytes. (a) Lentiviral-transduced ReN cell lines were produced that overexpress the mouse, hamster and human 129M and 129V versions of PrP^C^. Lysate from proliferating ReN progenitor cell lines was western blotted for PrP^C^ using the 6H4 (recognizes mouse and human PrP) and 3F4 (recognizes hamster and human PrP) monoclonal antibodies. Total protein signal was measured in Bio-Rad TGX stain-free gels according to manufacturer’s instructions. (b) the 129M and WT ReN cell lines exhibit a similar pattern of protein expression for PrP, TUBB3 and GFAP throughout the process of differentiation into neurons and astrocytes. ReN cells were differentiated as thin-3D cultures and lysate collected at weekly timepoints post-differentiation was western blotted for PrP (via 3F4 mAb), TUBB3 and GFAP. Signal was normalized to the most prominent band in the corresponding total protein image (see arrow). (c) Normalized protein expression of PrP, TUBB3 and GFAP in ReN WT and 129M cells throughout standard differentiation. Signal intensity was quantified using imageJ and normalized to the signal from the strongest band identified in total protein images. (d) qPCR-based quantification of GFAP, TUBB3 and PRNP of ReN 129M, WT and Empty cells over 4 weeks of differentiation. PRNP was quantified using PCR primers that bind the non-coding mRNA region (PRNP) and protein-coding region of the mRNA (PRNP_CDS). * *p*-value<0.05 (calculated using one-way ANOVAs).

Consistent and stable overexpression of PrP^C^ is an important consideration for developing *in vitro* models of prion replication. Lentivirus transduction proved effective for delivering PrP^C^ transgenes and producing ReN cell lines that express different versions of PrP^C^. Prior to lentivirus transduction, we employed CRISPR-mediated knockout to disrupt the sequence of *PRNP* in the wildtype ReN cell line. This resulted in a ReN VM *PRNP*^*-/-*^ cell line (denoted here ReN KO) that is deficient in endogenous PrP^C^ expression and was subsequently transduced with different lentivirus constructs. Puromycin antibiotic selection was next used to enrich for successfully transduced cells and establish stable cell lines (Figure 2a,b). Finally, successful transduction was confirmed via fluorescence microscopy of GFP expression in lentivirus-transduced ReN cells (Figure 2c). According to this method, we produced ReN cell lines that express the mouse, hamster, human-129 M and human-129 V versions of PrP^C^ (Table 1). To confirm expression of the appropriate version of PrP^C^, we western blotted lysate from each cell line (grown as proliferating neural progenitor cells) using the 6H4 and 3F4 monoclonal antibodies, which recognize human/mouse PrP and human/hamster PrP, respectively (Figure 3A). We found that the monoclonal antibodies recognized PrP^C^ from the appropriate cell lines and that the transduced cells overexpressed PrP^C^ compared to the WT cells. The ReN 129 M and 129 V cell lines had very similar levels of PrP^C^ expression (Figure 3a). We attributed differences in signal between the mouse, hamster and human PrP overexpressing cells to differences between antibody-epitope binding affinities, and could not distinguish true differences in protein abundance.

**Table 1.** Summary of ReN cell lines. WT – wild type; KO – PRNP knocked out; NPC – neural progenitor cell; PuroR – puromycin resistance; GFP – green fluorescence protein.

ReN Cell line	Parent cell line	PrP sequence	PrP expression	Transgene
WT	Primary NPCs	Human 129MV	PrP^+/+^	None
KO	WT	N/A	PrP^−/−^	None
Empty	KO	N/A	PrP^−/−^	EF1-(empty)-PGK-*GFP*-T2A-*PuroR*
Mmu	KO	Mouse	PrP^++/++^	EF1-(mmu)*PRNP*-PGK-*GFP*-T2A-*PuroR*
Ham	KO	Hamster	PrP^++/++^	EF1-(ham)*PRNP*-PGK-*GFP*-T2A-*PuroR*
129M	KO	Human 129M	PrP^++/++^	EF1-(129M)*PRNP*-PGK-*GFP*-T2A-*PuroR*
129V	KO	Human 129V	PrP^++/++^	EF1-(129V)*PRNP*-PGK-*GFP*-T2A-*PuroR*

To identify any effects of lentiviral transduction on ReN cell differentiation, we also compared protein and RNA expression of PrP, TUBB3 and GFAP longitudinally between ReN WT and 129 M cells throughout 4 weeks of differentiation (Figure 3c,d). In the case of qPCR, we additionally examined the ReN Empty cell line. Semi-quantitative analysis of western blot and qPCR data revealed increased TUBB3 abundance in all cell lines beginning at day 7 post differentiation (Figure 3c,d), consistent with differentiation into neurons and astrocytes. The overall pattern of TUBB3 and GFAP expression was similar between the WT and 129 M cell lines throughout differentiation, although we noted that GFAP abundance decreased consistently in 129 M cells compared to WT (Figure 3c,d). We also confirmed continuous PrP^C^ overexpression throughout differentiation in ReN 129 M cells and found that PrP^C^ expression increased over time post-differentiation in ReN 129 M and WT cells (Figure 3c,d).

**Figure 4.**
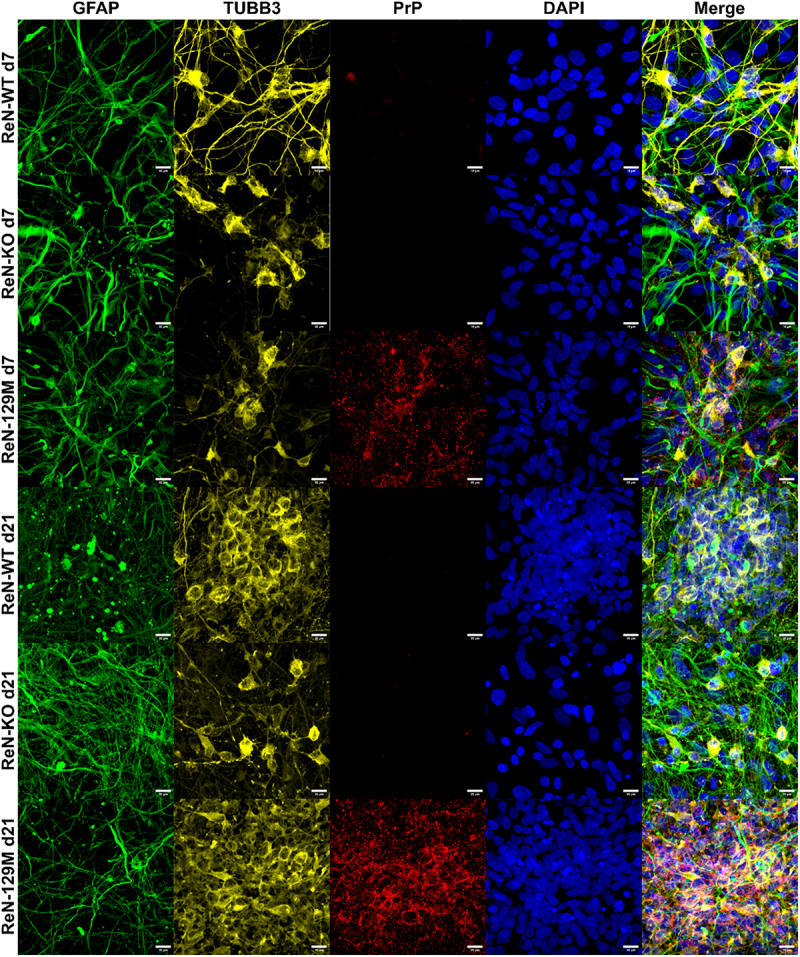
PrP^C^ is localized to TUBB3 expressing neurons in ReN 129M cells. Immunofluorescence was used to image ReN WT, KO and 129M cells by staining for TUBB3 (yellow), GFAP (green) and PrP (Red). Nuclei were counterstained with DAPI (Blue). Representative images are shown at days 7 and 21 post-differentiation. Images were acquired using the 63X oil immersion objective of a Zeiss LSM 700 instrument (scale bar = 10 µm).

To characterize PrP^C^ localization in lentivirus-transduced ReN neurons and astrocytes, we stained ReN WT, KO and 129 M cells for PrP, TUBB3 and GFAP and visualized them via immunofluorescence at day 7 and day 21 post-differentiation (Figure 4). Due to the weak expression of PrP^C^ in the WT cells, we failed to detect any PrP signal in these cells via immunofluorescence. However, we detected PrP in the 129 M cells and observed increased expression on day 21 compared to day 7 of differentiation, consistent with the western blot and qPCR data. When the thin-3D method of differentiation was employed (Figure 1c), we noticed that the ReN cells arranged into 3D spheroid-like clusters of TUBB3^+^ neurons separated by a monolayer of GFAP^+^ glial cells (assuming enough cells are seeded on the coverslip). PrP^C^ staining was highly associated with these spheroids of TUBB3^+^ neurons in the 129 M cells, and the staining pattern suggested localization to the cell membrane (Figure 4). Conversely, little PrP^C^ signal was detected in GFAP^+^ astrocytes. Thus, we concluded that lentiviral transduction primarily resulted in PrP^C^ overexpression within TUBB3^+^ neurons of differentiated ReN cultures.

### PrP^C^ expression is regulated during ReN cell differentiation and modulates the formation of spheroid-like structures by TUBB3^+^ neurons

The motivation for examining differentiation of ReN 129 M, WT and KO cells was to identify any unwanted effects from lentiviral transduction that could influence prion replication. As such, a comprehensive evaluation of the effects of PrP^C^ expression on the phenotype or function of ReN cells was beyond the scope of this study. However, we noticed regulation of PrP^C^ during ReN cell differentiation and observed modulated formation of spheroid-like structures by ReN cells that express different levels of PrP^C^. We report these findings because they are consistent with the role of PrP^C^ as a regulator of neurogenesis and neuron differentiation [35].

First, we noticed that protein abundance of PrP^C^ steadily increased throughout standard differentiation in both ReN 129 M and WT cells (Figure 3c). We also observed increased abundance of *PRNP* mRNA throughout differentiation in both ReN WT and empty cells, but not 129 M cells (Figure 3d). These differences in *PRNP* RNA expression pattern between ReN 129 M and WT cells are likely explained by *PRNP* being under the control of the EF1 promoter in the lentiviral-transduced 129 M cells. We also noticed that slower migrating species of PrP^C^ were lost upon differentiation in both ReN 129 M and WT cells, whereas faster migrating species became predominant (Figure 3b). The faster PrP^C^ migration pattern could imply that the di-glycosylated form of PrP^C^ is lost upon differentiation, or alternatively might correspond to truncation of PrP^C^. The increased *PRNP* RNA abundance, increased PrP^C^ protein abundance, and altered PrP^C^ migration pattern throughout ReN cell differentiation imply regulation of PrP^C^ at the transcriptional, translational, and post-translational levels, respectively.

**Figure 5.**
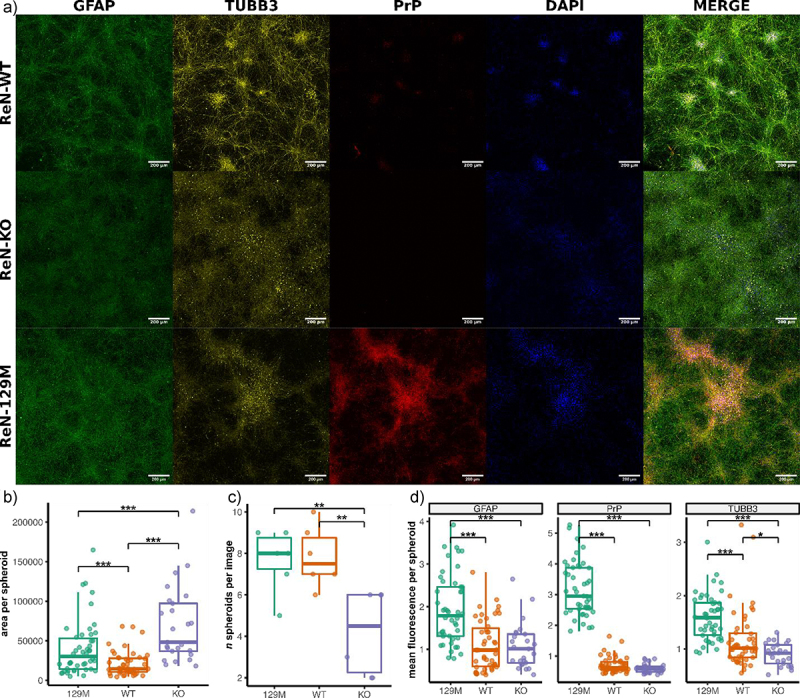
Altered arrangement into spheroid-like structures by ReN cells that express different levels of PrP^C^. (a) Representative Z-stack projections of 5x5 tile images taken with a 20x objective of ReN 129M, WT and KO cells after staining for TUBB3, GFAP and PrP at day 21 post-differentiation (scale bar = 200 µm). Area and fluorescence intensity within individual spheroids were analyzed with imagej. Fluorescence of GFAP, PrP and TUBB3 was normalized to the fluorescence of DAPI. (b) Area per spheroid. (c) Number of spheroids per image. (d) Mean normalized fluorescence intensity per spheroid. * p-value < 0.05, ** p-value < 0.01, *** p-value < 0.001. p-values were calculated using one-way ANOVAs.

We also visualized the size and number of 3D spheroid-like structures formed by the ReN 129 M, WT and KO cell lines that differed in their level of PrP^C^ expression. Spheroids were visualized at day 21 post-differentiation using 5 × 5 tile Z-stacked images taken of ReN cells stained for TUBB3, GFAP and PrP (Figure 5a). We found both ReN 129 M and KO cells to form larger spheroids compared to WT (Figure 5b). ReN empty cells formed fewer spheroids compared to ReN 129 M and WT cells (Figure 5c), and we noticed that while spheroids formed by the ReN KO cells took up a larger horizontal area compared to WT and 129 M, they did not occupy as much of a vertical distance within the Z-stacked images. We concluded that the ReN KO cells were deficient in forming spheroids compared to ReN WT and 129 M cells. When examining fluorescence signal intensity, we found ReN 129 M spheroids to express higher levels of GFAP, PrP and TUBB3 compared to WT and KO (Figure 5d). Altogether our results suggest that ReN cells with higher levels of PrP^C^ expression may form larger spheroids that have higher expression of TUBB3. However, we noticed weaker localization of TUBB3 to neurites with more signal originating from cell bodies in the ReN KO and 129 M compared to WT cultures, making the spheroid-like structures more easily visible in the WT cell line. This raises the possibility of differences between the ReN WT, KO and 129 M cell lines being influenced by selection during the CRISPR and lentivirus transduction steps and/or increased passage number.

### PrP^C^ overexpressing ReN cells do not replicate prions

ReN cultures that overexpress PrP^C^ within TUBB3^+^ neurons might offer a promising paradigm for *in vitro* prion replication. In initial attempts at optimizing this system, we did not observe convincing differences related to prion replication when comparing 2D-monolayer versus thin-3D differentiated cultures, or when inoculum was added at day 0 versus day 7 of differentiation (supplementary figure S4). We ultimately decided to use the thin 3D method of differentiation because a larger number of cells can be used (more lysate for PrP^Sc^ detection assays) and because these cultures can be maintained longer (more time to accumulate replicated PrP^Sc^). These thin 3D cultures were inoculated on day 7 of differentiation to allow the cells time to recover from the initial shock of growth factor removal.

**Figure 6.**
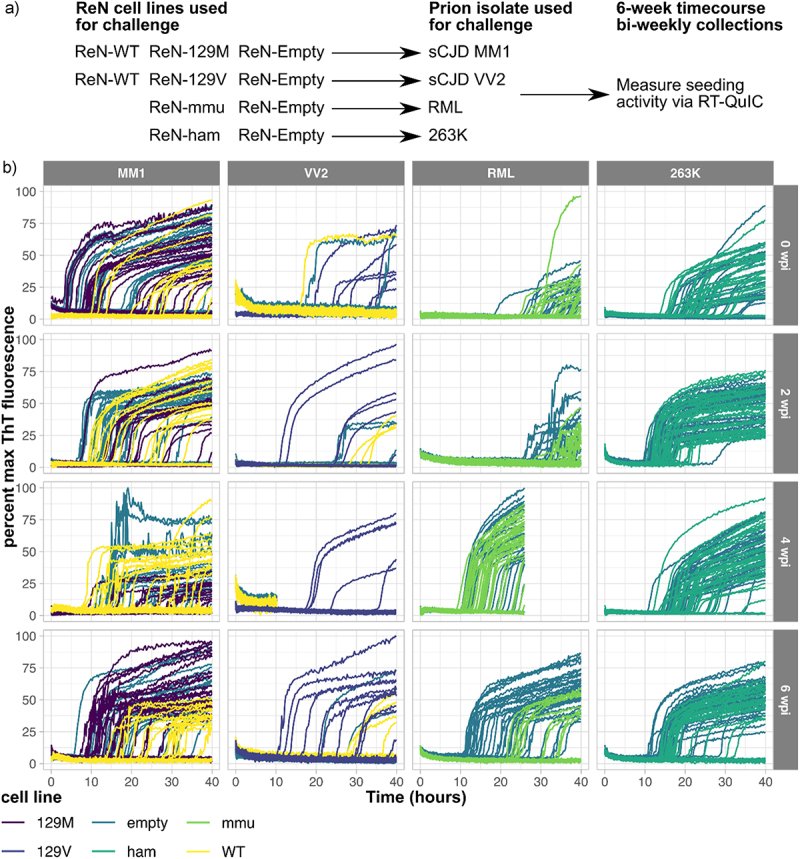
Prion seeding activity is consistently detected within ReN cultures for 6 weeks following inoculation with four prion isolates. To examine prion replication in differentiated ReN cultures, ReN-PrP^−/−^, ReN-PrP^+/+^ and ReN-PrP^++/++^ cells were differentiated for one week, then inoculated with one of four prion isolates. Prion inocula included human clinical isolates of Scjd-MM1 and Scjd-VV2, the RML strain of mouse-adapted scrapie, and the 263K strain of hamster-adapted scrapie. ReN cultures were maintained for 6-week post-inoculation with lysate collections in triplicate every 2 weeks. (a) Schematic representation of the experimental workflow. PrP^Sc^ amyloid seeding activity in ReN cell lysates was measured via RT-QuIC at each time point post-inoculation with the four prion isolates. (b) ThT fluorescence signal is plotted against reaction time for RT-QuIC assays seeded with 5e-08, 5e-09 and 5e-10 g total protein of ReN cell lysate.

To assess prion replication in this system, we performed four different prion challenge experiments of ReN cells with sCJD MM1 and sCJD VV2 human prion isolates, as well as RML and 263K rodent adapted scrapie (Figure 6a). CJD inocula was sourced from clinical cases identified by the Canadian CJD Surveillance System and was selected because they represented typical cases of sCJD MM1 and sCJD VV2. PrP^Sc^ amyloid seeding activity within inocula was characterized and quantified via RT-QuIC (supplementary figure S5). Each culture made from approximately 2 × 10^6^ ReN cells was exposed to 3.2 × 10^5^, 5.1 × 10^3^, 5.1 × 10^6^ and 5.1 × 10^6^ SD_50_ amyloid seeding equivalents of sCJD MM1, sCJD VV2, RML and 263K, respectively. Prion replication over time was assessed by measuring PrP^Sc^ amyloid seeding activity in lysate (detected via RT-QuIC) collected from the cells at bi-weekly time points out to 6 weeks post infection. RT-QuIC reactions were seeded in quadruplicate with 5 × 10^−8^, 5 × 10^−9^ and 5 × 10^−10^ grams total protein of ReN cell lysate. We detected positive seeding activity through increased ThT fluorescence over reaction time (40 h) across all of the conditions tested (Figure 6b). ReN cells treated with MM1 and 263K inoculum generally eliciting stronger RT-QuIC signal compared to those treated with RML and VV2 inoculum – likely attributed to differences between the amounts of PrP^Sc^ present in the different inoculums used. Indeed, amyloid seeding activity within VV2 inoculum was approximately 2 logs lower compared to the other isolates (supplementary figure S5), explaining the inefficient seeding activity in ReN cells after challenge with sCJD VV2.

**Figure 7.**
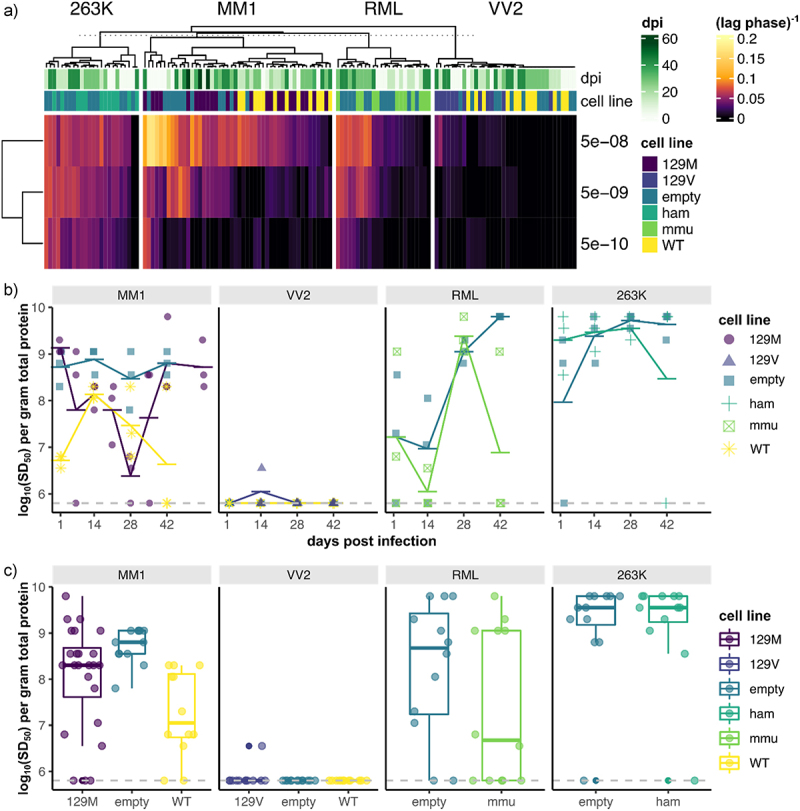
Prion seeding activity within ReN cultures does not surpass residual inoculum throughout 6-weeks post challenge with four prion isolates. PrP^Sc^-seeding activity was quantified via lag-phase^−1^ measurements from each individual RT-QuIC reaction. The mean lag-phase^−1^ value for each dilution (5e-8, 5e-9 and 5e-10 g total protein of lysate) per sample was then visualized as a hierarchical-clustered heatmap (a). RT-QuIC lag-phase^−1^ measurements did not cluster based on timepoint post-inoculation, suggesting that prion-seeding activity did not change over time in any of the conditions tested. We applied the spearman-karber transformation to the RT-QuIC data to obtain a single quantitative measurement of prion-seeding activity within each sample, expressed as log10(SD_50_) per gram of total protein. To assess prion replication, RT-QuIC log10(SD_50_) was plotted against days post infection (b), and to compare overall seeding activity per condition, RT-QuIC log10(SD_50_) was plotted per each cell line and inoculum (c). Prion seeding activity in ReN-PrP^+/+^ and ReN-PrP^++/++^ cells never surpassed ReN-PrP^−/−^ cells, which were included to account for signal from residual inoculum.

To get a better sense of quantitative differences in amyloid seeding activity between cell lines and over time, we performed hierarchical clustering of average lag phase^−1^ measurements for each sample (Figure 7a). While we observed differences in lag phase^−1^ measurements between inoculums used, we did not observe any clear clustering of samples that would suggest differences between cell lines or changes over time post-inoculation. We also employed the Spearman-Karber transformation to obtain a robust quantitative measurement of amyloid seeding activity (SD_50_) within each sample. While we again observed quantitative differences in amyloid seeding activity between inoculums used, we did not see convincing evidence of prion replication over time (Figure 7b) or any differences in SD_50_ measurements overall between cell lines used (Figure 7c). Importantly, the amyloid seeding activity within PrP^C^ overexpressing cells (ReN 129 M, 129 V, mmu and ham) never rose above that seen in the PrP^C^-deficient cells (ReN empty). From this, it is apparent that the amyloid seeding activity detected via RT-QuIC corresponds to residual inoculum present within the cultures for the entire duration of the 6-week time course. The detection of PrP^Sc^ in ReN cell lysate implies successful exposure of cells to the inoculum, and so we concluded that ReN cultures do not promote prion replication under the conditions tested.

## Discussion

We report that the differentiated cultures of ReN cells were resistant to infection with multiple prion isolates despite a high level of PrP^C^ expression mediated through lentivirus transduction. Specifically, through repeated measurements of amyloid seeding activity in 6-week time course experiments, we failed to observe replication of MM1 and VV2 type sCJD, or RML and 263K rodent adapted scrapie. Our findings are striking because others have reported that similar cultures derived from differentiated neural progenitor cells can be infected with prions [17–24]. The lack of PrP conversion we observed in this paradigm is also counterintuitive, given the capacity of ReN cells to replicate misfolded proteins in models of Alzheimer’s disease [25–28]. We were careful to verify the expression of PrP^C^ and confirm proper differentiation of the lentiviral-transduced ReN cell lines, finding PrP^C^ to be highly expressed by TUBB3^+^ neurons following differentiation. Although we did note an effect of PrP^C^ expression on the formation of 3D spheroid like structures in the differentiated ReN cultures, consistent with the role of PrP^C^ as a regulator of neurogenesis [35]. Nonetheless, had the ReN cultures successfully replicated prions, a system like this would be useful for characterizing a wide range of prion strains – providing a particularly valuable platform when applied to clinical isolates of human prion diseases.

We detected residual inoculum within the ReN cell cultures for as long as 6 weeks post-exposure (Figure 7), illustrating a major challenge of prion research – distinguishing *de novo* prion replication from PrP^Sc^ present within the inoculum. Here, we designed our study to distinguish *de novo* prion replication by comparing PrP^C^-overexpressing with PrP^C^-deficient ReN cell lines, and so we can conclude with some certainty that the ReN cultures did not promote prion replication. We consistently detected PrP^Sc^ of residual inoculum throughout the entire duration of the time course, irrespective of the inoculum used (sCJD MM1, sCJD VV2, RML or 263K). These findings are supported by a recent study showing that infectivity of sCJD MM1 prions can persist indefinitely in the brain throughout the lifespan of *Prnp*^*-/-*^ mice [36]. Thus, our findings reinforce that the longevity of residual inoculum-derived PrP^Sc^ must be carefully considered when modelling prion replication.

PrP glycosylation is an important factor for prion replication, given that PrP^Sc^ glycosylation differences can be used to distinguish prion strains [37,38] and that host PrP^C^ glycosylation can influence the outcome of prion infection [39,40]. We noticed that ReN cells expressed predominantly faster migrating PrP^C^ species upon differentiation (Figure 3b), perhaps indicating that mono-glycosylated and non-glycosylated forms were primarily available for PrP^Sc^ replication. Although we did not extensively characterize PrP^C^ glycosylation here, which could confirm whether differentiated ReN cultures completely lack di-glycosylated PrP^C^, the apparent changes in PrP^C^ glycoform ratios are worth considering in the context of prion replication. Many studies report an association between decreased glycosylation of PrP^C^ with enhanced prion replication. For instance, glycosylation deficiency of PrP^C^ can enhance cell-free conversion of PrP^Sc^ [41], enhance PrP aggregation and toxicity *in vitro* [42], and promote spongiform degeneration and plaque formation *in vivo* [43], while glycosylation deficiency of PrP^Sc^ is a consistent feature of prion pathogenesis [38]. Another study showed differential prion propagation of PrP glycosylation mutants, but the authors were unable to discern between amino acid mutations and glycosylation mutations [44]. Altogether, there is agreement that di-glycosylation of PrP^C^ can stabilize the structure and protect against misfolding. Therefore, a lack of di-glycosylated PrP^C^ expressed by differentiated ReN cells in theory would make this system more suitable towards prion replication.

Glycosylphosphatidylinositol (GPI) anchoring of PrP^C^ and localization to the cell surface on lipid rafts is another important post-translational modification that can influence prion replication. Membrane-associated GPI-anchored PrP^C^ is required for prion replication *in vitro* [45] and is required for neuroinvasion and neuronal spread of PrP^Sc^ *in vivo* [46]. Additionally, lack of sialylation on PrP^C^’s GPI anchor resulted in delayed disease onset *in vivo* [47]. In the differentiated ReN cultures, the immunofluorescence staining pattern of PrP was consistent with membrane localization in TUBB3^+^ neurons (Figure 4), indicating that at least some PrP^C^ was membrane-associated and therefore GPI-anchored. However, we did not characterize the composition of PrP^C^’s GPI-anchor in the differentiated ReN cultures, which might have yielded additional insights into the lack of prion replication observed.

It is also possible that certain aspects of our approach to culturing ReN cells had a negative impact on prion replication. For instance, in some circumstances, antibiotics can inhibit prion replication [48,49]. We supplemented ReN cell media with Gibco Antibiotic-Antimycotic to prevent contamination throughout the 6-week time course experiments that followed challenge with different prion inoculums. This yielded concentrations of 0.1 mg/mL penicillin, 0.1 mg/mL streptomycin, and 250 ng/mL Amphotericin B. Streptomycin were shown to inhibit prion replication at a concentration of 1.0 mg/mL but not 0.5 mg/mL [48], and so we would not expect 0.1 mg/mL streptomycin to have any effect. Several studies have shown Amphotericin B to prolong the incubation period of prion disease in various rodent models of scrapie infection [50–52]. When applied to *in vitro* cultures of scrapie-infected GT1–7 and N2a cells, Amphotericin B delayed PrP^Sc^ accumulation yet failed to prevent prion infection when used at a relatively high concentration of 4500 ng/mL [53]. A different study found Amphotericin B to reduce PrP^Sc^ levels in N2a cells chronically infected with 22 L scrapie when the concentration was 5000 ng/mL and above, but not at 2500 ng/mL [54]. To the best of our knowledge, penicillin is not known to inhibit prion infection. Therefore, we deemed it unlikely that the use of antibiotics prevented prion replication in this study, although we cannot completely rule out the possibility of antibiotics having a negative impact. Additionally, the composition of the extracellular matrix is known to be a major determinant of permissiveness to prion replication *in vitro* [55]. Here, the differentiated ReN cell cultures were embedded in Matrigel, and it is possible that this was not an ideal matrix composition for prion replication. However, findings by others argue against this hypothesis. Matrigel-embedded ReN cells accumulated misfolded proteins in an Alzheimer’s disease model [25–28], and replication of human prions was reported with stem cell-derived astrocytes cultured on Matrigel [56] and Matrigel-embedded cerebral organoids [57]. We also supplemented the ReN culture media with heparin – a glycosaminoglycan that was shown to promote *in vitro* prion replication [58]. Altogether we cannot conclude that our cell culture approach was responsible for the lack of prion replication observed.

In conclusion, overexpression of PrP^C^ was not enough to confer differentiated ReN cultures susceptible to infection with sCJD MM1, VV2, RML or 263K prions. While we could not determine the explanation for the resistance of ReN cells to prion infection, our findings reflect the current lack of any immortalized human cell line that are permissive to infection with naturally occurring human prion isolates [10]. It remains a significant challenge to develop novel *in vitro* models of prion infection that are capable of replicating a range of prion strains including those in humans, are susceptible to prion-induced toxicity, and are amenable to genetic manipulations.

## Methods

### ReN cell culture

ReN VM neural progenitor cells (Millipore-Sigma cat. no. SCC008) were proliferated in complete growth medium that consisted of DMEM/F12 medium supplemented with N21 supplement (Millipore-Sigma Cat. No. SCM081), 20 ng/mL bFGF (ThermoFisher Cat. No. PHG0368), 20 ng/mL EGF (ThermoFisher Cat. No. PHG0313) and 0.45 U/mL Heparin (Millipore-Sigma Cat. No. H3149-10KU). Matrigel (Corning Cat. No. 354234)-treated cell culture vessels containing pre-warmed growth medium were seeded with ReN cells and cultured at 37°C and 5% CO_2_. To maintain growth, the complete volume of growth medium was exchanged 2–3 times per week. ReN cells were passaged at confluence by first rinsing with PBS, dissociating with Accutase (Millipore-Sigma) at 37°C for 2–5 min, and diluting the cells 1:5 in growth medium. Accutase was removed by pelleting the cells via centrifuging at 100 ×g for 3 min and re-suspending the pellet in growth medium before seeding a fresh Matrigel-treated culture vessel. ReN cells were passaged up to a maximum of 15 times before a new vial of cells was thawed from liquid Nitrogen storage.

### 2D standard differentiation

ReN cells were seeded and cultured in complete growth medium for 2–3 days until they reached confluence. The complete volume of growth medium was then exchanged with differentiation medium that lacked growth factors (DMEM/F12 supplemented with N21 and 0.45 U/mL Heparin). ReN cells were differentiated over several weeks by replacing the differentiation medium every 2–3 days. Morphological changes coinciding with differentiation were tracked using a phase-contrast microscope.

### Thin 3D standard differentiation

Proliferating ReN cells were dissociated with Accutase, pelleted, re-suspended in cold differentiation medium, mixed 1:1 with ice-cold Matrigel, and then diluted further 1:11 with cold differentiation medium (final Matrigel dilution of 1:12). This mixture was added to culture dishes, incubated at 37°C for 1 h, and then topped up with an equal volume warm differentiation medium. For 12-well plates, 2 × 10^6^ cells were seeded per well. For coverslips, 1.5 × 10^5^ cells in 150 uL Matrigel mixture were seeded on 12 mm coverslips, forming a droplet that was incubated at 37°C for 1 h before topping up with 400 µL differentiation medium. Cultures were maintained at 37°C by exchanging media with fresh differentiation medium three times per week.

### Preparation of ReN VM PRNP-/- (KO) cell line

ReN *PRNP* KO cells were generated by transfecting wildtype proliferating ReN cells with *S. pyogenes* Cas9 and *PRNP*-targeting gRNA with the protospacer sequence AACACCGGTGGAAGC at a ratio of 3:1 using EditPro (GlobalStem MTI) according to manufacturer’s protocol, in proliferation media without Heparin. On day 2 post-transfection, cells were passaged to generate single-cell colonies that were later expanded for genomic analysis. To verify *PRNP* KO, ReN cell genomic DNA was purified using QIAamp DNA Mini Kit (cat no. 51304, Qiagen, Valencia, CA, USA), and 50 ng of genomic DNA were amplified with the Q5 Hot Start High-Fidelity 2× PCR Master Mix (cat no. M0494L, NEB) using primers 5’-TCTTTGTGACTATGTGGACTG-3’ and 5’-TGCCACATGCTTGAGGTTGGTT-3’. The PCR products were column purified and sequenced on an ABI PRISM 3100 Genetic Analyzer.

### Preparation of lentivirus

For production of lentivirus to overexpress PrP^C^, the commercially available lentiviral transfer plasmid pCDH-EF1-MCS-(PGK-copGFP-T2A-Puro) (Systems Biosciences cat. no. CD813A–1; referred to herein as pCDH813A) was used. pCDH813A includes a GFP fluorescent reporter protein and a resistance marker to the antibiotic puromycin. Open reading frame (ORF) sequences encoding human 129 M, human 129 V, mouse and hamster versions of PrP^C^ were cloned into pCDH813A in front of the EF1 promoter using the BamHI and NotI restriction sites. Sanger sequencing was used to confirm the successful insertion of each PrP^C^ sequence into pCDH813A.

Lentiviral vectors were produced using the Lentistarter 3.0 kit (Systems Biosciences) according to manufacturer’s instructions. Briefly, ~70% confluent 150 mm dishes of HEK-293T cells were treated with a transfection mixture containing 4 µg of the corresponding pCDH813A lentiviral transfer plasmid, 45 μL pPACKH1 plasmid mix and 55 μL PureFection transfection reagent in 1.6 mL of serum-free DMEM. The transfection mixture was vortexed for 10 s and incubated at room temperature for 15 min before adding dropwise across the 150 mm dish with HEK-293Ts. Lentivirus-containing media (20 mL) was collected from the 150 mm dish at 48 and 72 h following transfection and cleared by centrifuging at 1,000 ×g for 15 min. Lentivirus was then precipitated from the media mixing with 5 mL of PEG-it, incubating at 4°C for 24 h and centrifuging at 1,000 ×g for 15 min. The lentivirus-containing pellet was re-suspended in ice-cold PBS and stored in aliquots at −80°C.

### Lentiviral transduction of ReN cells

ReN *PRNP*^−/−^ cells (ReN KO) were transduced with lentivirus by diluting the concentrated lentiviral preparation 1:600 in 15 mL complete growth medium that was added to the ReN cells at~30%-50% confluence in T75 flasks. The ReN cells were left with the lentivirus mixture for 72 h before passaging the cells. Puromycin then was used to select for transduced ReN cells by treating with 0.5 μg/mL puromycin in complete growth medium. The cells were cultured with 0.5 μg/mL puromycin until they reached confluence, at which point liquid nitrogen stocks were prepared.

### Western blotting

Cells were collected with a cell scraper in PBS, pelleted and washed 3× in PBS, re-suspended in 100 µL PBS with protease inhibitor, and sonicated via 2 × 30 s bursts at low intensity. Debris was then cleared from the lysate by centrifuging at 14,000 *xg* for 10 min. Lysate was diluted in laemmli buffer (BioRad) with 5 mM DTT (Millipore-Sigma) and boiled for 5 min before electrophoresis. 20 μg of lysate per well was then loaded onto 10% or 8–16% TGX Stain-free SDS-PAGE (BioRad) and electrophoresis was run at 300 V. Total protein was imaged on a BioRad Gel dock imager according to the ‘Stain Free’ protocol prior to transfer to PVDF low-fluorescence membranes using the BioRad Transblot system. Membranes were blocked with 5% skim milk diluted in TBST for 1 h at room temperature before incubating with primary antibodies in blocking buffer at 4°C overnight. The membranes were then washed 3× for 5 min with TBST before incubating with secondary antibodies in blocking buffer at room temperature for 1 h. The membranes were washed 4× for 5 min with TBST and visualized using the Pierce femto-sensitivity ECL substrate kit (ThermoFisher), or via fluorescence depending on the secondary antibodies used.

**Antibodies**: 6H4-mouse-anti-PrP (1:2,000, ThermoFisher 7,500,996), 3F4-mouse-anti-PrP (1:2,000, Millipore Sigma MAB1562), rabbit-anti-GFAP (1:100,000, DAKO Z0334), mouse-anti-TUBB3 (1:5,000, Abcam ab78078), rabbit-anti-NeuN (1:2,500, Abcam ab177487), rabbit-anti-NES (1:2,000, Abcam ab105389), rabbit-anti-SYN (1:2,500, Abcam ab64581), goat-anti-mouse-IRDye-800 (1:5,000, LI-COR 925–32210), goat-anti-rabbit-IRDye-680 (1:10,000, LI-COR 925–68021), goat-anti-mouse-HRP (1:5,000, DAKO P0447) and goat-anti-rabbit-HRP (1:5,000, DAKO P0448).

### Immunofluorescence

Cells were fixed with 4% PFA for 1 h at room temperature and washed 3× with PBS. Triton-X 100 at 0.5% in PBS was used to permeabilize the cells for 1 h at room temperature. Cells were then blocked with 10% goat serum+1% BSA in PBST for 1 h at room temp before incubating with primary antibodies in blocking buffer overnight at 4°C. Cells were washed 4× for 5 min with PBST and then were incubated with secondary antibodies in blocking buffer for 4 h at room temperature. Cells were washed 6× for 5 min in PBST, incubated with 300 mM DAPI for 1 h at room temperature, rinsed 3× in PBS and then coverslips were mounted using Prolong Glass with NucBlue. Images were acquired with a Ziess LSM 700 confocal microscope.

To visualize ReN spheroids, 5 × 5 tile Z-stacked images were taken. Three representative images were taken of two separate cover slips for each cell line (*n = 6*). Spheroid number, area, GFAP signal, PrP signal and TUBB3 signal were determined using imagej.

**Antibodies**: rabbit-anti-NES (1:250, Abcam ab105389), mouse-anti-TUBB3 (1:300, Abcam ab78078), rabbit-anti-GFAP (1:1,000, DAKO Z0334), chicken-anti-PrP (1:300, Abcam ab178545), goat-anti-rabbit-Alexa488 (1:1,000, Abcam ab6150077), goat-anti-mouse-Alexa568 (1:250, ThermoFisher A11004), goat-anti-chicken-Alexa647 (1:250, ThermoFisher A32933).

**qPCR**RNA was extracted from the ReN cells using the Qiagen RNeasy mini kit according to manufacturer’s instruction, and then quantified using a Nanodrop 1000. cDNA was then prepared from 300 ng total RNA using the ThermoFisher High Capacity cDNA synthesis kit in 10 µL reactions of 10 min at 25°C, 120 min at 37°C and 5 min at 85°C. 2 µL of the cDNA reaction was then supplied as template for TaqMan qPCR in 20 µL reactions made with the TaqMan Fast Universal PCR master mix according to manufacturer’s instructions. The following TaqMan gene expression assays were used: ACTB (Hs03023943_g1), GFAP (Hs00909233_m1), TUBB3 (Hs00801390_s1), PRNP (Hs01920617_s1) and PRNP_CDS (Hs04937277_s1).

### Prion infection of ReN cells

ReN cells were differentiated as thin-3D cultures for 7 days prior to inoculation. Brain homogenate (10%) was diluted 1:500 into differentiation medium and exchanged with ½ the volume of medium covering the ReN cells (1:1000 final dilution of 10% brain homogenate = 0.01%). Cells were incubated with the inoculum for 48 h prior to completely exchanging medium, after which the cells were maintained by exchanging ½ the volume of differentiation medium (supplemented with Gibco Antibiotic-Antimycotic) three times per week. At the indicated time points, cells were collected with a cell scraper, pelleted and washed 1× with cold PBS, re-suspended in 100 µL PBS, homogenized via 2 × 30 sec sonication bursts and then total protein content in lysate was quantified using Pierce BCA assays.

In total, four different inoculums were used: sporadic Creutzfeldt-Jakob disease (sCJD) type MM1 and VV2, as well as RML mouse-adapted scrapie and 263K hamster-adapted scrapie. Inoculum was prepared as 10% brain homogenate in PBS via 2 × 30 s sonication bursts and clarified via two 10 min centrifugations at 2,000 *xg*. Whole brains used to make RML and 263K inocula were collected at clinical endpoint of disease from a CD1 mouse and Golden-Syrian hamster, respectively. CJD inocula were prepared from cortical tissue taken from two cases of typical sCJD MM1 and sCJD VV2 that were identified by the Canadian CJD Surveillance System (CJDSS) and were genotyped and glycotyped according to standard diagnostic methods. The human tissue was approved for use in research with appropriate consent under Health Canada-Public Health Agency of Canada Research Ethics Board reference number REB 2017-009P. These inocula have been used in an unrelated, ongoing *in vivo* mouse experiment and display the expected amount of infectivity (J. Myskiw et al, unpublished data).

### RT-QuIC

Lysate was serially diluted 10-fold in PBS supplemented with 0.04% SDS and 0.4% N2 supplement (Gibco). RT-QuIC reactions were prepared in 96-well plates by mixing 5 μL of diluted lysate with 95 μL of a master mix to give reactions with 300 mM NaCl, 1 mM EDTA, 10 nM ThT, 0.002% SDS and 10 μg substrate PrP in PBS. Full-length hamster (29–231) PrP substrate was prepared in-house via recombinant expression in bacteria followed by purification with histidine affinity chromatography according to published methods [59]. In standard assays, sample dilutions of 5 × 10^−8^, 5 × 10^−9^, and 5 × 10^−10^ grams total protein were used to seed RT-QuIC reactions (*n = 4* technical replicates). Each RT-QuIC plate included brain homogenate from a 263K and mock-infected hamster as positive and negative technical controls, respectively. The 96-well plates were sealed with optical adhesive film and run on FLUOstar Omega microplate readers (BMG) in 15-min cycles of double-orbital shaking followed by ThT fluorescence measurements (450 nm excitation and 480 nm emission) for a maximum of 50 h. We processed raw fluorescence data with MARS software (BMG) and exported in `.csv` format for plotting and further analysis in RStudio.

## Supplementary Material

Supplemental Material
