# Supplementary material for: Differentiated cultures of an immortalized human neural progenitor cell line do not replicate prions despite PrPC overexpression
Source: Prion. 2023 May 2;17(1):116–32. doi: 10.1080/19336896.2023.2206315 (PMC10158546; doi:10.1080/19336896.2023.2206315)
Supplement: Supplemental Material [file KPRN_A_2206315_SM8957.docx]

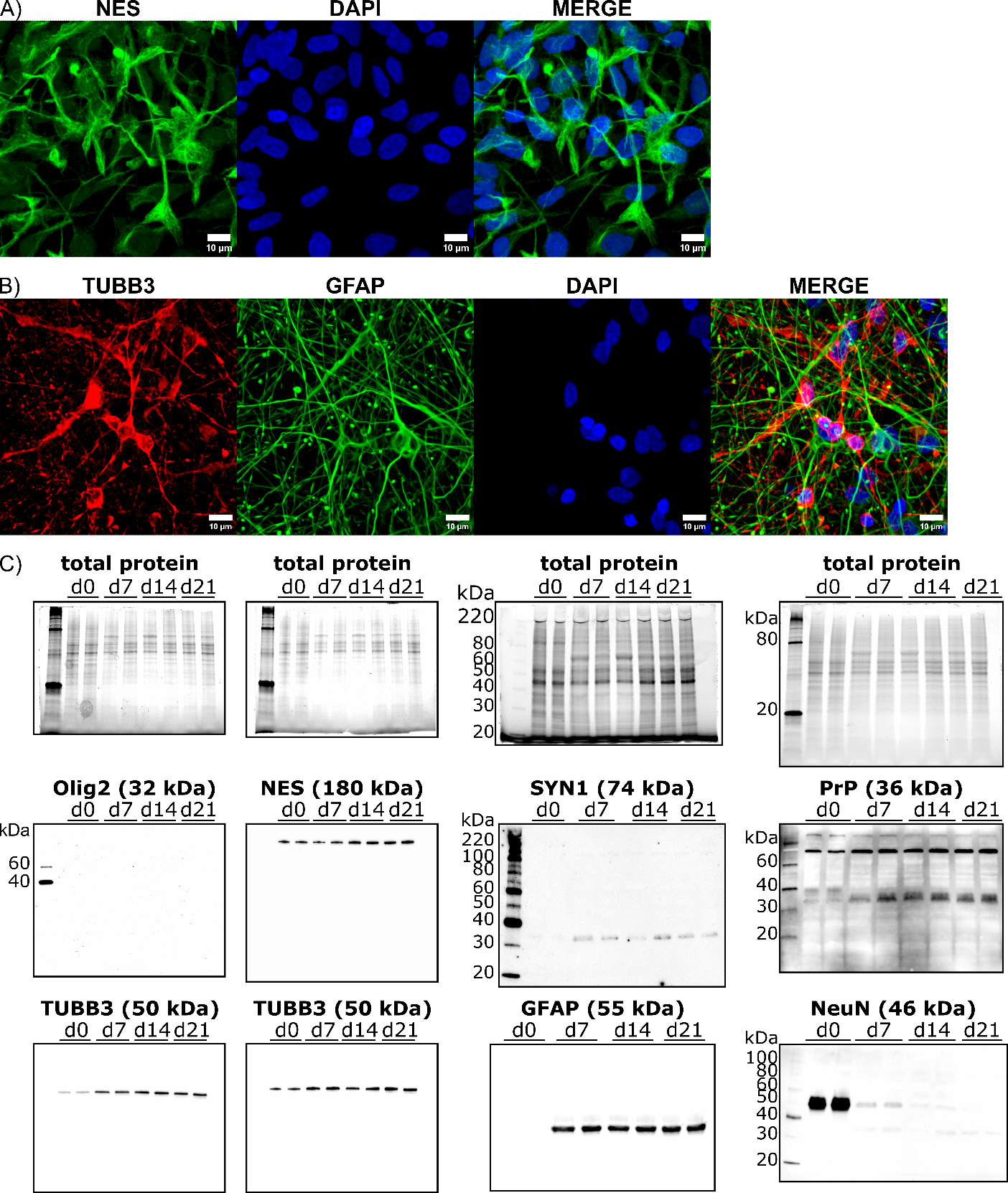


**Supplementary Figure 1. Accompanying uncropped images to Figure 1.** A) Immunofluorescence of NES in ReN neural progenitor cells as day 0 of differentiation. B) Immunofluorescence of TUBB3 and GFAP in ReN cultures at day 28 of differentiation. Immunofluorescence images were acquired using the 63X oil immersion objective of a Zeiss LSM 700 instrument (scale bar = 10 µm). C) Complete images showing western blotting of lysate from ReN cells at days 0, 7, 14 and 21 of differentiation.


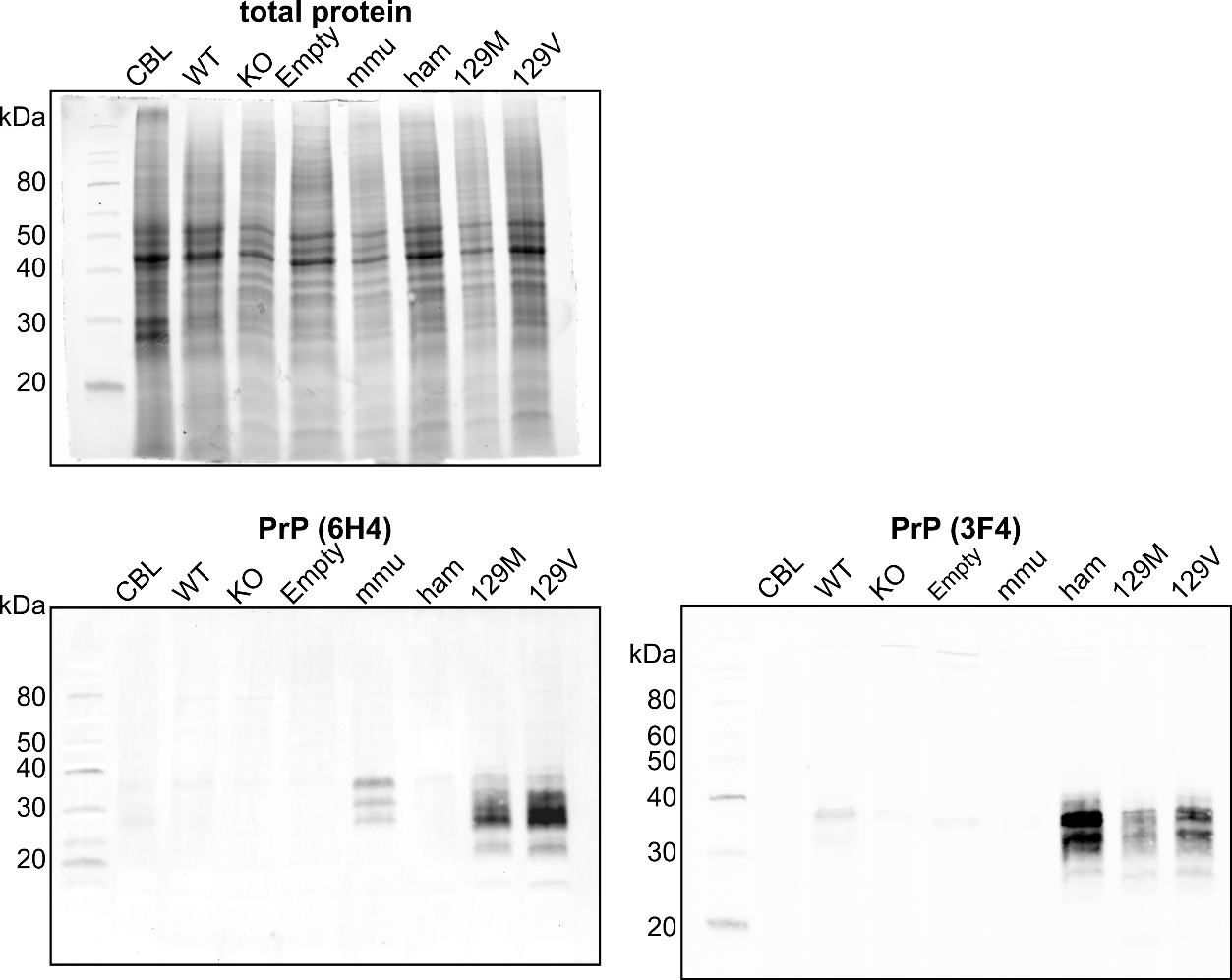


**Supplementary Figure 2. Uncropped western blot images from Figure 3A.** Lysate from proliferating ReN cell lines was blotted using the 6H4 and 3F4 monoclonal antibodies against PrP.

**
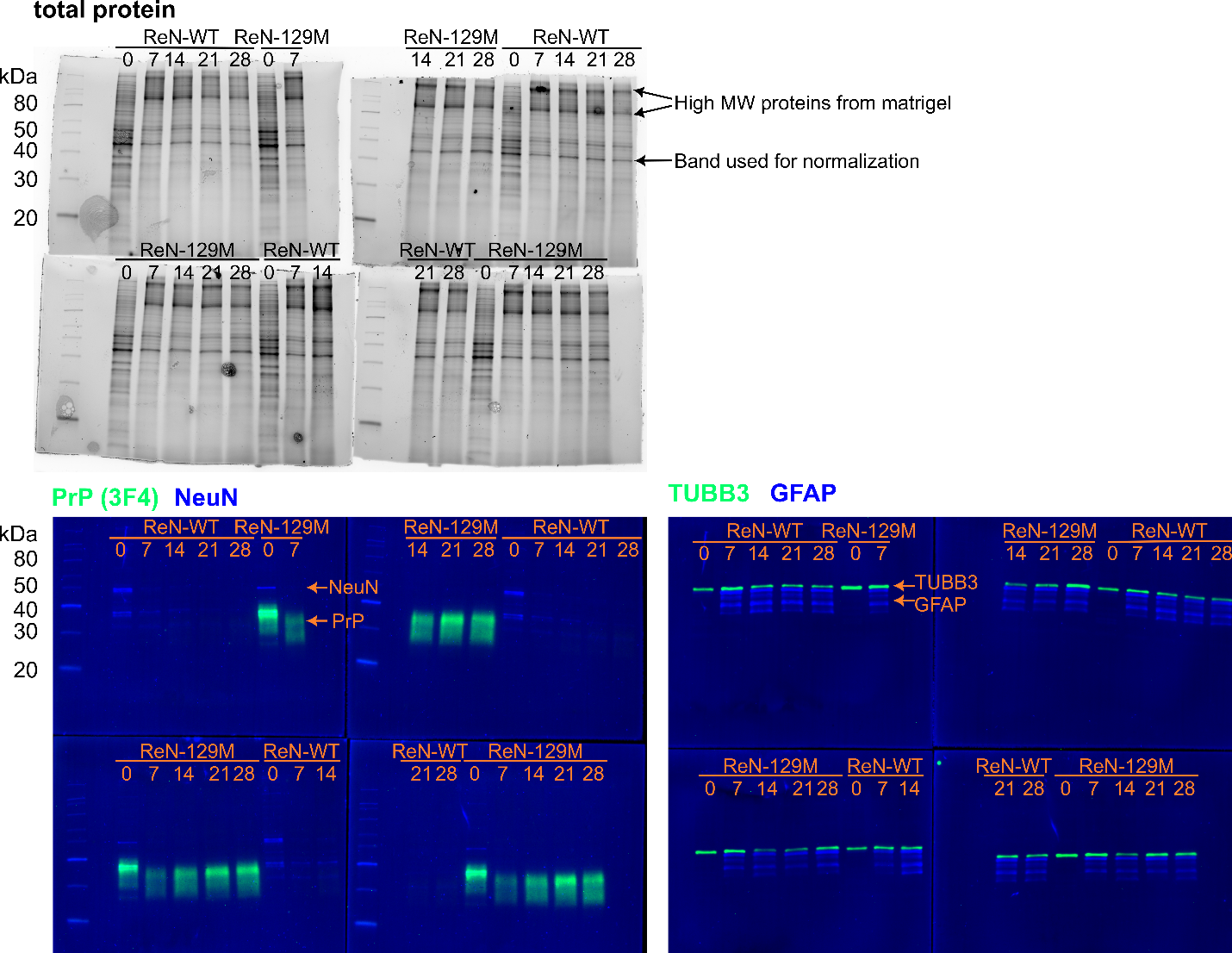
**

**Supplementary Figure 3. Uncropped western blot images from Figure 3B.** Lysate was collected from ReN-WT and ReN-129M cells at days 0, 7, 14, 21, and 28 of differentiation in triplicate, and western blotted for PrP, NeuN, TUBB3 and GFAP.


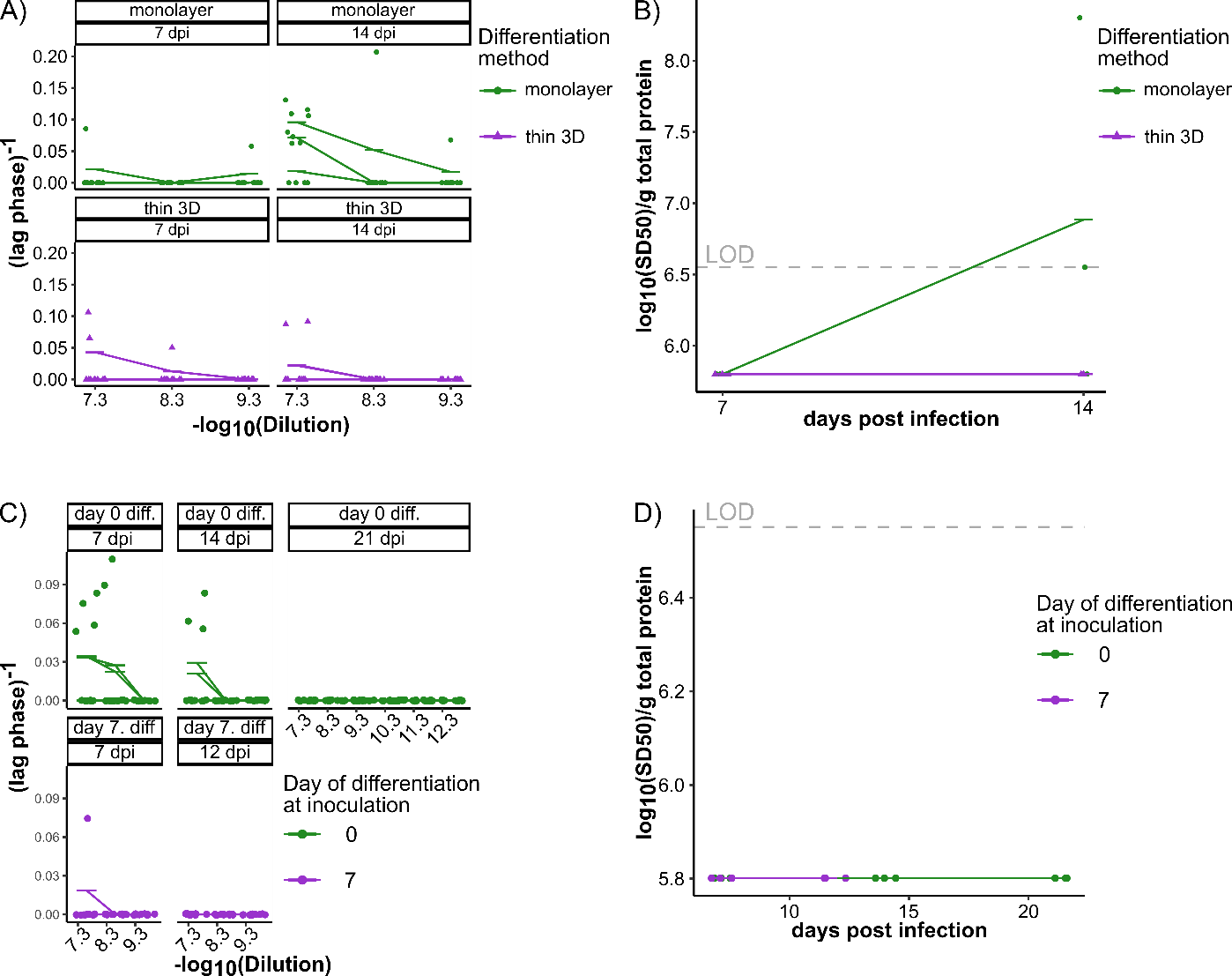


**Supplementary Figure 4. Attempts to optimize replication of RML prions in ReN cultures.** A) RT-QuIC lag phase^-1^ measurements and B) SD_50_ measurements of lysate collected from ReN-mmu cells following challenge with RML inoculum as either monolayer or thin-3D differentiated cultures. Amyloid seeding activity was assessed at days 7 and 14 post infection. C) RT-QuIC lag phase^-1^ measurements and D) SD_50_ measurements of lysate collected from ReN-mmu cells following challenge with RML inoculum at day 0 or day 7 of differentiation. Amyloid seeding activity was assessed at days 7, 14, and 21 post infection for cultures that were inoculated on day 0 of differentiation, and at days 7 and 12 post infection for cultures that were inoculated at day 7 of differentiation.


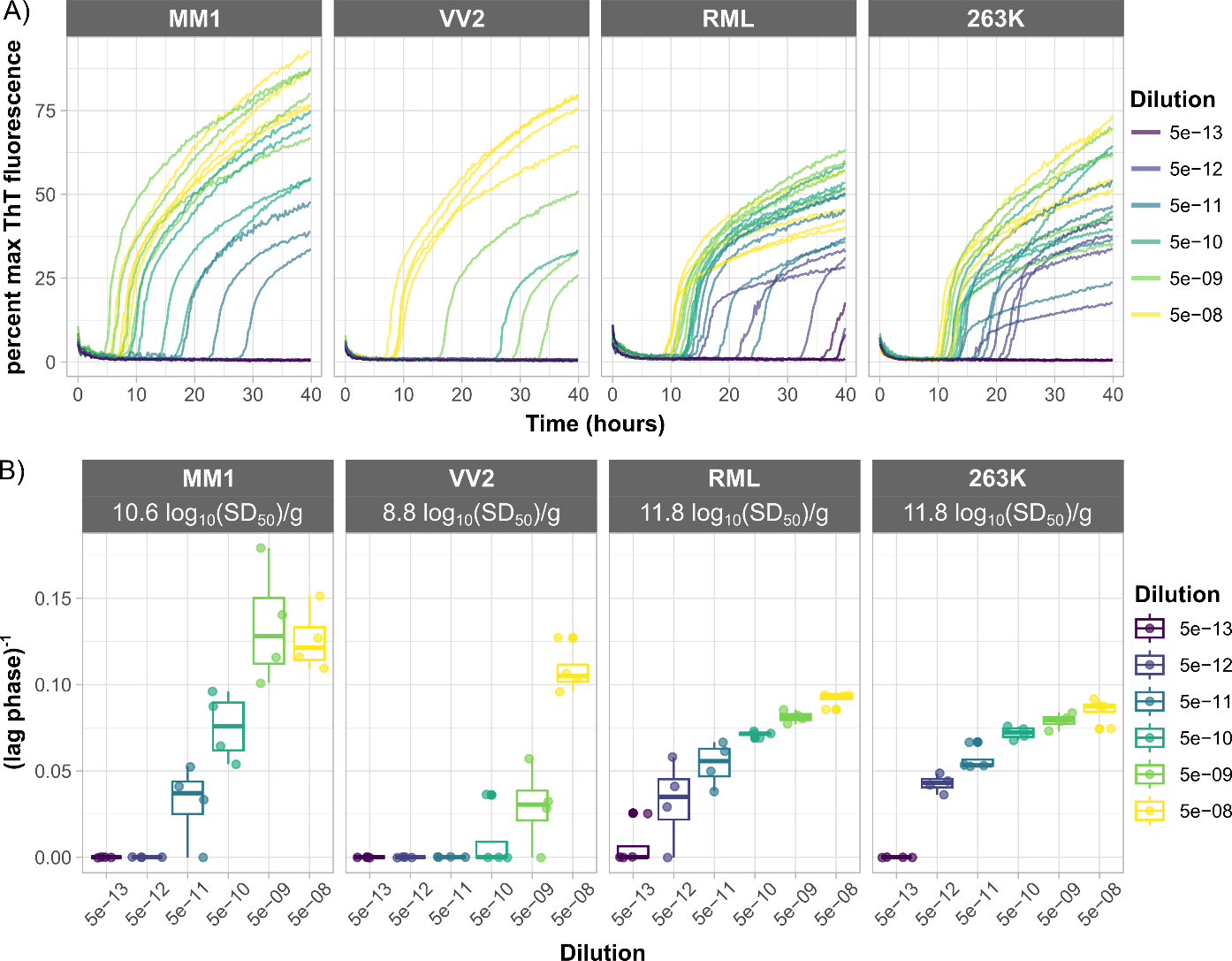


**Supplementary Figure 5. Prion amyloid seeding activity within inocula used to challenge ReN cultures.** RT-QuIC was used to measure the total amyloid seeding activity within each inoculum used to challenge the ReN cultures including clinical isolates of sCJD MM1 and sCJD MM2, and RML and 263K rodent adapted scrapie. (A) ThT fluorescence signal is plotted against RT-QuIC reaction time for assays seeded with 5e-08, 5e-09, 5e-10, 5e-11, and 5e-12 grams total protein of inoculum. (B) PrP^Sc^-seeding activity was quantified via lag-phase^-1^ measurements from each individual RT-QuIC reaction and is plotted per dilution. The total amyloid seeding activity is indicated for each inoculum, calculated as log_10_(SD_50_) per gram total protein as per the Spearman-Karber transformation.
